# Nonsurgical Management of Oral Mucocele by Intralesional Corticosteroid Therapy

**DOI:** 10.1155/2016/2896748

**Published:** 2016-10-16

**Authors:** Rupam Sinha, Soumyabrata Sarkar, Tanya Khaitan, Arpita Kabiraj, Anirban Maji

**Affiliations:** ^1^Department of Oral Medicine and Radiology, Haldia Institute of Dental Sciences and Research, Purba Medinipur, Haldia, West Bengal 721645, India; ^2^Department of Oral Pathology & Microbiology, Haldia Institute of Dental Sciences and Research, Purba Medinipur, Haldia, West Bengal 721645, India

## Abstract

*Background*. Oral mucocele is a common lesion resulting from an alteration of minor salivary glands due to mucus accumulation. Rapid appearance, specific location, history of trauma, bluish colour, and consistency help in the diagnosis. Conventional surgical removal is the treatment of choice but has several disadvantages like damage to adjacent ducts with further development of satellite lesions. Therefore, the present study was undertaken to evaluate the efficacy of intralesional corticosteroid injection (betamethasone) as a nonsurgical treatment procedure in oral mucoceles.* Material and Method*. A total of 20 cases (males and females, 10–30 years of age) with clinically diagnosed oral mucoceles were given 1 mL of betamethasone intralesionally. All the patients were examined after a period of 7, 14, and 21 days to evaluate the response of the lesion towards treatment and consequently given the 2nd, 3rd, 4th injections. If the lesion resolved after one or two injections, the treatment was discontinued.* Results*. Out of the 20 cases, 18 of them showed complete regression of the lesion whereas the remaining 2 cases showed decrease in size. All the patients received maximum of 4 consecutive shots in weekly interval.* Conclusion*. Intralesional corticosteroid therapy can be considered as the first choice in the treatment of oral mucoceles.

## 1. Introduction

Mucoceles are most common benign lesions of the oral cavity developing as a result of retention or extravasation of mucus material from the minor salivary glands. They are derived from Latin words “muco” and “coele,” meaning mucus and cavity, respectively; henceforth by definition, mucus filled cavities. They represent the 17th most common lesion of oral cavity with an incidence of 2.5 lesions per 1000 patients, frequently in the second decade of life and rarely among children under one year of age [1, 2].

Mucoceles are broadly classified into two types: extravasation and retention type. Extravasation mucocele results from a traumatized salivary gland duct with consequent spillage into the soft tissues around the gland whereas retention type appears due to a decrease or absence of glandular secretion produced by blockage of the salivary gland ducts [2]. They are benign soft tissue masses clinically characterized by single, painless, soft, smooth, spherical, and translucent and fluctuant nodule, which is usually asymptomatic [3].

There are various treatment modalities which include surgery, laser ablation, cryosurgery, sclerotherapy, micromarsupialization, laser surgery, and intralesional injection of sclerosing agent or corticosteroid [3]. Although surgery is widely used, it has several disadvantages such as lip disfigurement and damage to adjacent ducts with further development of satellite lesions [4]. However, usage of intralesional corticosteroid is meagre in the literature. Luiz et al. (2008) and Baharvand et al. (2014) reported cases treated with intralesional corticosteroids whereas Mortazavi et al. (2014) had attempted combined intralesional dexamethasone and micromarsupialization [5–7]. Therefore, considering this background the present study was undertaken to evaluate the efficacy of intralesional corticosteroid injection (betamethasone) as a nonsurgical treatment procedure in oral mucoceles.

## 2. Material and Method

The study was initiated after the protocol had been approved by the Institutional Committee of Research Ethics. All the subjects were being informed about the importance of the study and written informed consent was obtained for the same. A total of 20 cases (males and females) within an age range of 10 to 30 years with clinically diagnosed oral mucoceles attending the outpatient department of oral medicine were recruited in the study. Patient with any history of contraindications for systemic steroids and those not willing to receive injections were excluded from the study.

**Figure 1:**
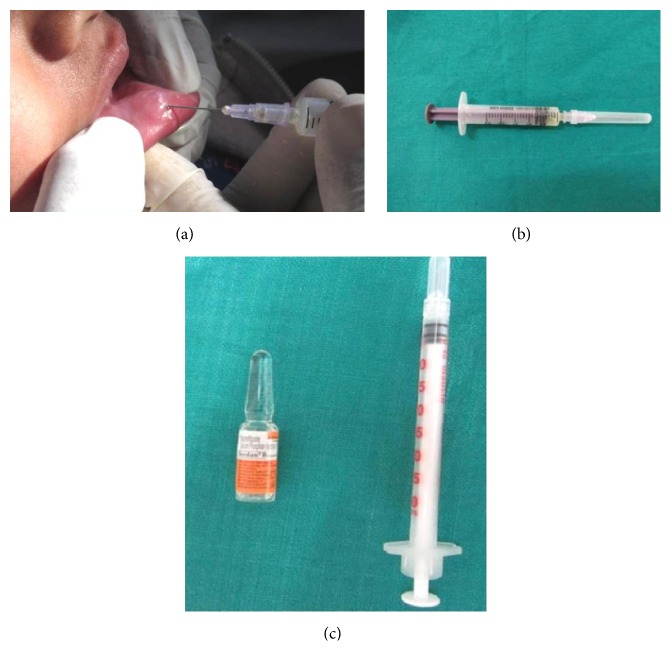
(a) Mucus aspirated by 18-gauge needle and syringe, (b) syringe with mucus aspirate, and (c) 1 mL of betamethasone intralesional injection and 31-gauge insulin syringe.

All the subjects were principally diagnosed based on the following clinical features: location, history of trauma, rapid appearance, variations in size, bluish color, and consistency [1]. Firstly, surface local anaesthesia was applied and mucus aspirated with the help of 18-gauge needle and a syringe. Then 1 mL of betamethasone (4 mg/1 mL) was slowly injected by insulin syringe (0.3*∗*8 mm size, 31 gauges) to prevent any leakage, less discomfort, and pain (Figure 1). The solution was gradually injected into base of the lesion and adjacent to the periphery of the lesion.

All the patients were examined after a period of 7, 14, and 21 days to evaluate the response of the lesion towards treatment and consequently given the 2nd, 3rd, and 4th injections. If the lesion resolved after the first injection, the treatment was discontinued. The size of lesion was measured by means of a dental calliper in mm in weekly evaluation. After the completion of the treatment, the patients were evaluated after 1, 3, and 6 months to check for recurrence.

## 3. Results

A total of 20 clinically diagnosed cases of oral mucocele were selected for the study. The lower labial mucosa was found to be the most common site in the subjects (17 cases) followed by buccal mucosa (3 cases). All the subjects received 1 mL of betamethasone injection at 1-week interval till the lesion resolved. They were kept under weekly evaluation on days 7, 14, and 21 and periodic recall up to 6 months. The size of the lesion was measured with the help of dental calliper and varied from 5 to 20 mm.

**Figure 2:**
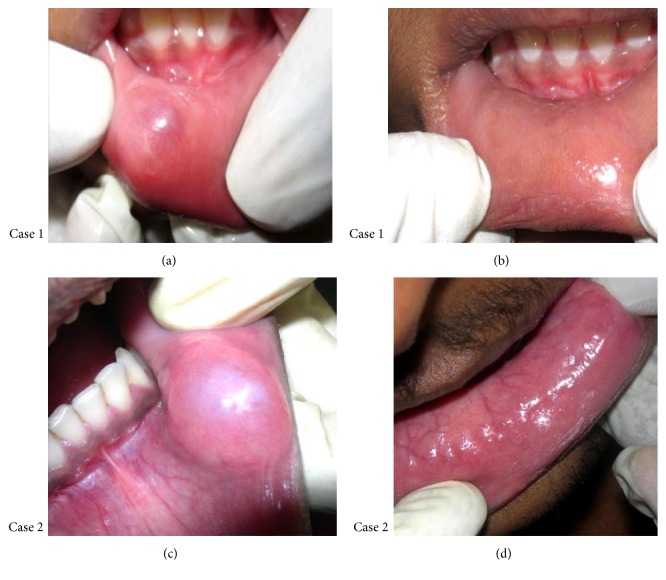
Case  1 showing mucocele in right lower labial mucosa measuring 5*∗*5 mm in size (a), complete regression of lesion after 2 intralesional injections (b), Case  2 showing large mucocele in left lower labial mucosa measuring 20*∗*15 mm in size (c), and complete regression of lesion after 4 intralesional injections (d).

**Figure 3:**
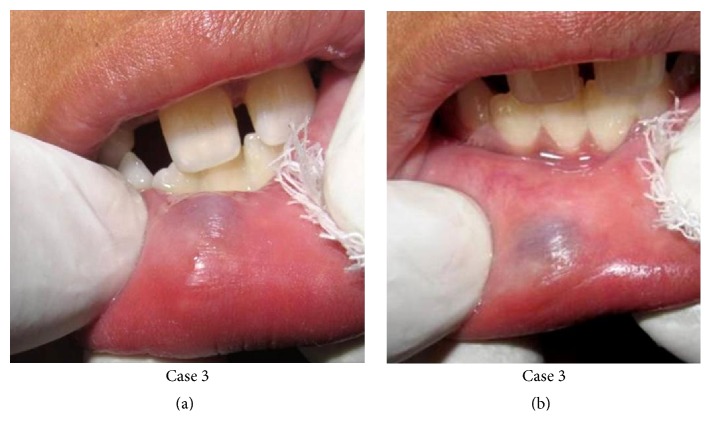
Case  3 showing mucocele in right lower labial mucosa of 10*∗*8 mm size (a) and decrease in size of lesion to 3*∗*2 mm (b).

Out of the 20 cases, 18 of them showed complete regression of the lesion whereas the remaining 2 cases showed decrease in size of the lesion (2-3 mm) (Figures 2 and 3). All the patients received maximum of 4 consecutive shots in weekly interval. No postoperative complications were observed except for minimal pain and local discomfort reported by few patients which resolved within an hour (Table 1).

## 4. Discussion

Mucocele is a self-limiting mucus containing cyst of salivary glands commonly occurring in the oral cavity, with relatively rapid onset and fluctuating size. They can also be encountered in the appendix, gall bladder, and lacrimal sac. Etiologic factors include trauma to the oral cavity such as lip biting, piercings, accidental rupture of salivary gland, and cheek biting or it may occur due to dilation of the duct secondary to its obstruction caused by a sialolith or dense mucosa. The pathogenesis of extravasation type occurs in three phases. In the first phase, there is spillage of mucin from salivary duct into the surrounding tissue in which some leucocytes and histiocytes are seen. In second phase, granulomas appear due to the presence of histiocytes, macrophages, and multinucleated giant cells associated with foreign body reaction followed by pseudocapsule formation in the last phase [1, 2, 8]. In retention type, obstruction of salivary gland duct leads to accumulation of salivary fluid into the duct, resulting in small balloon formation and as time progresses, the balloon increases in size and bulges into the oral cavity [9].

Mucocele frequently occurs in the second decade of life and has no gender predilection. The commonly affected sites are those that are most likely prone to mechanical trauma, that is, lower lip followed by tongue, buccal mucosa, and palate [3, 8]. In the present study, lower lip was seen to be the most common site (17 cases) followed by buccal mucosa (3 cases). Clinically they present as round, well circumscribed transparent bluish, soft cystic swelling varying in size from few millimetres to 3 cm. These lesions are usually asymptomatic, but they can cause discomfort and difficulty in speaking and chewing if they are abnormally large in size. The duration of the lesion is not constant and can last for few days to 3 years [1, 2, 8]. All these findings were in concordance with the subjects included in the present study.

Diagnosis of mucocele is mainly based on history and clinical appearance which includes rapid appearance, specific location, history of trauma, bluish color, soft consistency, and fluctuation [1, 9, 10]. Similar clinical diagnostic criteria were followed while conducting the study.

Mucoceles frequently resolve spontaneously. The decrease in size may be due to rupture of the lesion whereas subsequent mucin accumulation or reabsorption of saliva deposits may cause the lesion to reform. There are various treatment modalities which include surgical removal, CO_2_ laser ablation, cryosurgery, micromarsupialisation, marsupialisation, electrocautery, laser vaporization or laser surgery, and intralesional injection of corticosteroids or sclerosing agent. Micromarsupialisation has been proved to be a simple, relatively noninvasive, painless, effective, and low recurrence technique to treat oral ranulas and selected mucoceles by Amaral et al. (2012) and Sagari et al. (2012) wherein all cases showed complete healing within 30 days after the procedure [8, 11]. However, these surgery procedures have several disadvantages such as trauma, pain, lip disfigurement, damage to adjacent vital structures, and ducts leading to development of satellite lesions and can also be expensive to the patient [12, 13]. Therefore, we have undertaken a nonsurgical treatment protocol with highly potent corticosteroids (betamethasone). Corticosteroids act as the most potent anti-inflammatory agent inhibiting the expression of multiple inflammatory genes (encoding cytokines, chemokines, adhesion molecules, inflammatory enzymes, receptors, and proteins) and may also increase the transcription of genes coding for anti-inflammatory proteins including lipocortin-1, interleukin-1, and interleukin-10 receptor antagonist [14]. They also act like a sclerosing agent causing shrinkage of the dilated salivary ducts [6]. The present study was carried out in 20 oral mucocele patients treated with intralesional corticosteroid injections and complete regression in 18 cases was observed. The main objective of this procedure was to drain the mucus and reduce the size of the lesion.

Similar case series was reported by Baharvand et al. (2014) wherein seven cases treated with dexamethasone were cured totally and two showed reduction in size. No long standing complication was experienced postoperatively except for local discomfort reported by one [6]. Mortazavi et al. (2014) reported a large labial mucocele treated with combined intralesional dexamethasone and micromarsupialization which led to complete healing [7]. It was thus surveyed in our study that there was no uneasiness or irritation postoperatively (except for mild discomfort in few cases for short duration) and all the subjects were satisfied with the treatment procedure used.

## 5. Conclusion

Intralesional corticosteroid therapy is a good alternative nonsurgical procedure which can be performed in a short span of time, economical, esthetically more beneficial than surgery, cryotherapy, or laser ablation, and performed effortlessly. It is a relatively simple, repeatable, cost effective, and potentially curative method easily acceptable by the patient. To conclude, this treatment protocol can be considered as the first choice or substitute for surgery in the treatment of oral mucoceles and also be carried out in routine dental practice.

## Figures and Tables

**Table 1:** Distribution of cases in intralesional corticosteroid therapy in oral mucocele.

Case number	Location of lesion	Size of the lesion (mm)	Number of injections	Result	Complications
1	Lower labial mucosa	10*∗*10	4	Resolved	Nil
2	Lower labial mucosa	10*∗*8	4	Reduced in size (3*∗*2 mm)	Mild discomfort
3	Lower labial mucosa	6*∗*5	2	Resolved	Nil
4	Lower labial mucosa	20*∗*15	4	Resolved	Nil
5	Lower labial mucosa	5*∗*5	2	Resolved	Nil
6	Buccal mucosa	6*∗*5	3	Resolved	Nil
7	Lower labial mucosa	10*∗*10	4	Resolved	Mild discomfort
8	Lower labial mucosa	5*∗*5	3	Resolved	Nil
9	Lower labial mucosa	8*∗*5	4	Resolved	Nil
10	Buccal mucosa	5*∗*5	2	Resolved	Nil
11	Lower labial mucosa	10*∗*10	4	Resolved	Nil
12	Lower labial mucosa	5*∗*5	2	Resolved	Nil
13	Lower labial mucosa	10*∗*10	4	Resolved	Nil
14	Lower labial mucosa	5*∗*5	2	Resolved	Nil
15	Lower labial mucosa	15*∗*10	4	Reduced in size (2*∗*2 mm)	Nil
16	Buccal mucosa	5*∗*5	2	Resolved	Nil
17	Lower labial mucosa	10*∗*8	4	Resolved	Mild pain
18	Lower labial mucosa	8*∗*5	4	Resolved	Nil
19	Lower labial mucosa	10*∗*10	4	Resolved	Nil
20	Lower labial mucosa	12*∗*10	2	Resolved	Nil

*∗* denotes multiplication symbol.
